# Epileptic Seizure Detection Using Hyperdimensional Computing and Binary Naive Bayes Classifier

**DOI:** 10.3390/bioengineering12121327

**Published:** 2025-12-05

**Authors:** Xindi Huang, Hongying Meng, Zhangyong Li

**Affiliations:** 1 Department of Electronic and Electrical Engineering, Brunel University of London, London UB8 3PH, UK; xindi.huang@brunel.ac.uk; 2 Research Centre of Biomedical Engineering, Chongqing University of Posts and Telecommunications, Chongqing 400065, China; lizy@cqupt.edu.cn

**Keywords:** epileptic seizures detection, electroencephalogram, biomedical signal processing, hyperdimensional computing, binary Naive Bayes classifier

## Abstract

Epileptic seizure (ES) detection is critical for improving clinical outcomes in epilepsy management. While intracranial EEG (iEEG) provides high-quality neural recordings, existing detection methods often rely on large amounts of data, involve high computational complexity, or fail to generalize in low-data settings. In this paper, we propose a lightweight, data-efficient, and high-performance approach for ES detection based on hyperdimensional computing (HDC). Our method first extracts local binary patterns (LBPs) from each iEEG channel to capture temporal–spatial dynamics. These binary sequences are then mapped into a high-dimensional space via HDC for robust representation, followed by a binary Naive Bayes classifier to distinguish ictal and inter-ictal states. The proposed design enables fast inference, low memory requirements, and suitability for hardware implementation. We evaluate the method on the SWEC-ETHZ iEEG short-term dataset. In one-shot learning, it achieves 100% sensitivity and specificity for most patients. In few-shot learning, it maintains 98.88% sensitivity and 93.09% specificity on average. The average latency is 4.31 s, demonstrating that it is much better than state-of-the-art methods. These results demonstrate the method’s potential for efficient, low-resource, and high-performance ES detection.

## 1. Introduction

Epileptic seizure (ES) is a clinical manifestation of abnormal, excessive, and hyper-synchronous discharges in cortical neurons [1]. It can present with various symptoms such as uncontrollable convulsions, tremors, loss of consciousness, and blank stares [2,3], profoundly affecting patients’ daily lives. The most serious risk associated with epilepsy is its potential fatality, with mortality rates in adults reaching up to 3.6%. While anti-epileptic drugs effectively control seizures in about 70% of patients, the remaining 30% exhibit drug resistance [4], highlighting the urgent need for more accurate monitoring and timely intervention.

Electroencephalogram (EEG) records brain activity using electrophysiological indicators. It is closely associated with ES caused by abnormal brain discharges. ES detection relies on EEG signals for continuous monitoring of patients and rapid identification of seizure events [5]. This is a huge workload for neurologists due to the large number of patients whose seizures need to be detected [6]. Therefore, the development of automated ES detection methods plays a crucial role in supporting neurologists. These methods can help neurologists reduce missed or misdiagnosed cases [7]. In addition, automated ES detection methods help to analyze the frequency, duration, and pattern of seizures, providing valuable insights for developing a precise medication regimen or surgical treatment plan [8].

**Figure 1:**
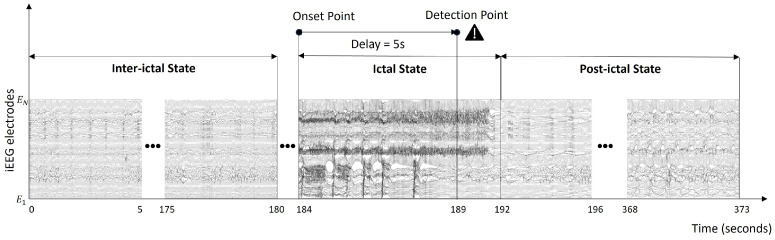
An EEG recording example from the SWEC-ETHZ iEEG short-term dataset [13] (e.g., the first seizure of patient 1). Three minutes from 0 s is labeled as the inter-ictal stage. The ictal stage is labeled from 184 to 192 s. The post-ictal stage is labeled from 192 to 373 s. The onset point is defined as the seizure onset annotated by neurologists and the detection point refers to the onset estimated by the proposed ES detection method. The detection delay is determined by the time difference between these two points. As the number of electrodes varies between patients, it is denoted by N in the figure.

For ES detection methods, the quality of EEG signal is crucial [9]. EEG signals are divided into intracranial EEG (iEEG) and scalp EEG (sEEG). iEEG is an invasive method where electrodes are implanted directly into the brain, offering significantly higher Signal-to-Noise Ratio (SNR) and fewer artifacts. This results in superior accuracy for ES detection and brain activity mapping. However, it involves surgical procedures like craniotomy, which carry risks such as long-term inflammation [10]. In contrast, sEEG is a non-invasive method that records EEG signals using electrodes placed on the scalp. This approach makes sEEG safer and more accessible. However, sEEG signals have lower SNR and are more prone to artifacts due to attenuation by the skull and surrounding tissues [11]. In clinical practice, EEG signals are analyzed in two main periods: ictal (during a seizure) and inter-ictal (the interval between seizures) [12]. ES detection aims to alert the patient when the onset point is detected. The time difference between the point detected by the algorithm and the point at which the seizure onset occurs is the detection delay. An example of ES detection applied to the iEEG signal is shown in Figure 1.

ES detection methods can be divided into two categories: deep learning and traditional machine learning. Deep learning-based ES detection methods have shown great potential, largely due to their ability to model complex spatio-temporal features of EEG signals [14]). Convolutional neural networks (CNNs) are commonly used to extract multi-scale spatial features from EEG signals [15,16,17,18]. On this basis, Xu et al. [19] proposed a multi-scale CNN model to shorten the latency of ES detection. Their method achieved 4.7 s detection latency and 0.08/h false detection rate in the SWEC-ETHZ iEEG short-term dataset. Long Short-Term Memory and transformer networks excel at capturing the inherent time-dependence of ES activity, providing directions for automatic ES detection [20,21]. Although deep learning methods have performed well in ES detection, their reliance on large-scale model parameter storage and high computational cost make them difficult to implement on wearable and low-power devices in real applications. Meanwhile, traditional machine learning, such as Support Vector Machines, Random Forests, and K-Nearest Neighbors [22] have been used for ES detection. Compared to deep learning methods, these methods usually require fewer parameters and less computational resources and data, making them more suitable for real-time ES detection in resource-limited environments [5].

As a traditional machine learning method for probabilistic inference, Bayesian theory [23] has been applied to ES detection since as early as 2009. Tzallas et al. [24] pioneered the use of the Naive Bayes classifier (NBC) for ES detection, laying the foundation for automated detection methods based on probabilistic reasoning. In recent years, several studies have also adopted the NBC for ES detection [25,26,27,28,29], further validating its feasibility across different datasets. However, these methods often require a larger amount of training data. These factors limit their generalizability in more complex scenarios. To solve these problems, hyperdimensional (HD) computing approaches have also garnered attention. Burrello et al. [30] introduced one-shot learning for iEEG ES detection using end-to-end binary operations, encoding iEEG signals with local binary pattern (LBP) features and mapping them into high-dimensional vectors. Detection was performed by comparing the Hamming distances of these vectors between inter-ictal and ictal periods. Later, they determined the epileptogenic zone by comparing the high-dimensional vector differences between different electrodes during ictal and inter-ictal periods [13]. In 2020, they combined three features, LBP, line length, and amplitude, to classify high-dimensional vectors by Hamming distance. The detection latency of this method on the SWEC-ETHZ iEEG short-term dataset was 8.81 s, which is the lowest value currently achievable by traditional machine learning methods [31]. However, the problems of low sensitivity, high average detection latency, and large high-dimensional vectors that are difficult to implement on hardware remain. Furthermore, while iEEG provides high-quality recordings, most existing algorithms are not suitable for real-time or embedded implementations due to high computational costs. In contrast, our approach is designed to meet the constraints of low-power, memory-constrained systems and can therefore be deployed in bedside monitors, implantable neurostimulators, or other future resource-constrained environments.

In summary, current methods for detecting ES through EEG signals still exhibit certain limitations. On the one hand, contemporary HD computational approaches typically suffer from significant detection delays and rely on large HD vectors that are difficult to implement efficiently in hardware. On the other hand, whilst iEEG can provide high-quality recordings, the substantial computational and memory costs involved render many existing algorithms unsuitable for real-time or embedded applications. In contrast, our method aims to develop and evaluate a lightweight, low-detection-delay, and high-detection-performance framework for ES detection.

## 2. Method

**Figure 2:**
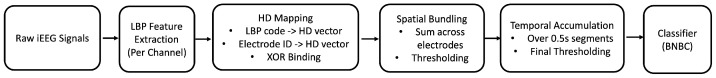
Overall architecture of the proposed ES detection framework. The raw iEEG signals are first encoded using a local binary pattern (LBP) for each channel. The LBP codes and corresponding electrode identifiers are projected into high-dimensional (HD) vectors and bound using XOR. These vectors are spatially bound and temporally accumulated to form the final HD representation, which is classified by a binary Naive Bayes classifier.

The overall architecture of the proposed ES detection framework is shown in Figure 2. This framework pioneers the integration of HD computation with a binary Naive Bayes classifier (BNBC) for detecting ES in iEEG signals. Specifically, our objective is to achieve reliable one-shot and few-shot learning performance while maintaining a lightweight model architecture.

**Figure 3:**
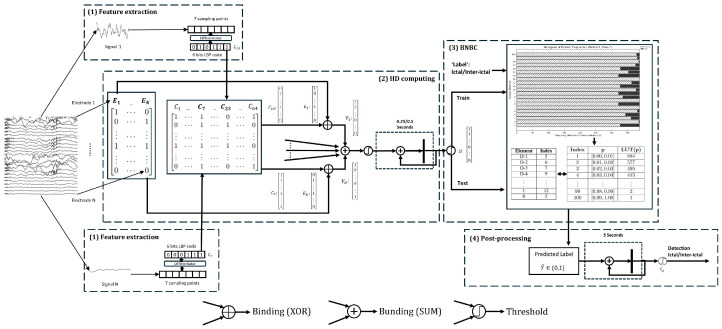
Overview of the proposed system: (1) Feature extract: A 6-bit LBP code is extracted from a 7-point segment for each electrode. (2) HD computing: HD computing is employed to transform these LBP codes and their corresponding electrodes into *D*-dimensional vectors. The LBP mapping matrix (*C*) encodes the spatial relationships of the LBP codes, while the electrode mapping matrix (*E*) aggregates the contributions of each electrode. LBP codes and corresponding electrodes are XOR-operated on every element. The results of the different electrodes are summed on an element-by-element basis and then processed using a threshold. These vectors are accumulated to form a vector *H*, representing the cumulative histogram of recordings for durations of 0.25 or 0.5 s. (3) BNBC: The BNBC first computes the histograms for the training data, capturing the distribution of features for both ictal and inter-ictal classes. These histograms are then mapped to a Lookup Table (LUT) for efficient retrieval during testing. During the testing process, the BNBC utilizes the LUT to classify the test data based on the learned distributions. (4) Post-processing: As a post-processing step, a patient-dependent voting mechanism ($t_{p}$) is implemented to aggregate the predictions over a 5 s window, ensuring a robust final classification decision for ictal or inter-ictal states.

Our proposed ES detection method consists of four main phases. Firstly, the feature extraction phase involves generating 6-bit LBP codes from 7 sampling points for each electrode, which effectively captures the local patterns of the EEG signals. Secondly, these LBP codes and corresponding electrodes are converted into high-dimensional vectors using the HD computing method, representing the signal features within time windows of either 0.25 s or 0.5 s. Thirdly, binary Naive Bayes classifier [32] is then applied to classify these high-dimensional vectors. During the training phase, the BNBC learns classification rules to distinguish between ictal and inter-ictal periods based on the high-dimensional feature representations. In the testing phase, the BNBC classifies the test signals as either inter-ictal or ictal. Finally, the detection decision is further optimized through a patient-dependent voting mechanism, which considers the results from the last 5 s of the prediction label. The detailed procedure of the method is illustrated in Figure 3.

### 2.1. Data Preparation

This study focuses on analysing the SWEC-ETHZ short-term iEEG dataset [13]. The dataset consists of 100 anonymized iEEG recordings from 16 patients with drug-resistant epilepsy, sampled at 512 Hz. Each recording consists of a 3 min pre-ictal period, an ictal period, and a 3 min post-ictal period, with the number of electrodes varying from 36 to 100. For pre-processing, the iEEG signal is first converted to a binary sequence using LBP coding. Each sample consists of seven sampling points, which are encoded by LBP to produce 6-bit binary values. LBP coding involves comparing the voltage amplitudes of two neighboring sample points: if the voltage at the latter sample point is greater than the voltage at the former, the binary value is assigned a value of 1; otherwise, it is assigned a value of 0. These 6-bit binary values are computed and stored as decimal numbers. The stored decimal numbers are then mapped into high-dimensional vectors.

### 2.2. Hyperdimensional Computing

To perform HD computing, we construct two mapping matrices whose elements are independently sampled from the normal distribution N(0,1). One matrix is used to map the coded LBP signals, whereas the other maps the corresponding electrodes into the same *D*-dimensional space.

Since the elements are independent and identically distributed, the resulting high-dimensional vectors are approximately orthogonal: for two independently sampled vectors $u,v\in R^{D}$, the expected inner product is $E[u^{⊤}v]=0$ and the variance of the normalized inner product decreases as 1/D. Thus, when D=1000, the cosine similarity between two random vectors concentrates tightly around zero with a standard deviation of approximately $1/\sqrt{D}\approx 0.03$, and the probability that two vectors exhibit a large correlation is negligibly small. In other words, different codes and electrodes are represented by quasi-orthogonal vectors in the HD space.

In our implementation, the LBP operator produces 6-bit codes for each signal sample. Each code therefore takes an integer value between 0 and 63, i.e., there are $2^{6}=64$ possible patterns. Consequently, we allocate 64 distinct high-dimensional vectors to represent the coded signals, each corresponding to 1 decimal value in the range 0–63. The number of high-dimensional vectors used for electrode mapping equals the number of electrodes available for a given patient; if a patient has *M* intracranial electrodes, we instantiate *M* electrode vectors and use only these rows of the electrode mapping matrix. This design naturally accommodates patients with different channel counts, while keeping all HD vectors in the same dimensionality *D*.

The LBP codes and their corresponding electrodes are then bound by applying a bit-wise XOR operation between the code vector and the electrode vector. The resulting bound vectors from different electrodes are bundled by element-wise summation across electrodes and processed by a majority threshold. Specifically, after summation, each dimension is compared to half of the number of bound vectors being aggregated: if an element exceeds this threshold, it is set to 1; otherwise, it is set to 0. The resulting binary vector represents the HD encoding of a single time step. These binary vectors are further accumulated over time to represent either 0.5 s or 0.25 s signal windows, followed by the same majority-thresholding operation. This hierarchical bundling process converts the raw iEEG signal into multiple binary high-dimensional vectors, each encoding a 0.5 s or 0.25 s interval, which are then used as input samples for subsequent classification.

There are several reasons for selecting 0.25 s and 0.5 s as the segmentation window sizes. The onset stage of ES typically occurs within low-to-mid frequency rhythms. The 0.25 s and 0.5 s window sizes can cover 1-2 cycles. This segmentation ensures real-time processing while providing essential temporal features. Secondly, throughout the framework, binding vectors require summation and thresholding operations. Excessively short windows may cause encoding fluctuations, while overly long windows significantly increase detection latency. Furthermore, when considering hardware deployment, shorter windows reduce the sample size and cache requirements per decision. Utilizing integer LUT and addition operations effectively lowers computational and storage demands.

### 2.3. One-Shot and Few-Shot Learning

Different training strategies were employed for various patients, classified based on the number of training seizures into one-shot learning and few-shot learning. In the one-shot learning approach, a single seizure along with its corresponding inter-ictal period was utilized for training, while the remaining seizures were reserved for testing. Conversely, the few-shot learning strategy involved training with multiple seizures and their corresponding inter-ictal periods with the remaining seizures set aside for testing. For a fair comparison with previous works [13,31], we used exactly the same learning strategies for these patients.

### 2.4. Binary Naive Bayes Classifier

Since the input samples are binary vectors, we employ the probabilistic method known as the BNBC [32], which is derived from Bayes’ theorem. In BNBC, the class descriptions of each feature vector in the training set are aggregated to construct a posterior probability model. During the testing process, the posterior probability that a given binary feature vector belongs to each category is computed. A key distinction of BNBC compared to traditional Bayesian classifiers is its ability to handle binary vectors, which significantly reduces computational complexity. This characteristic makes BNBC particularly suitable for large-scale or real-time applications.

In our method, given binary feature vectors $\left\{x_{k}\in \left\{0,1\right\},k=1,2,\ldots ,D\right\}$, the probability $P(x_{k}|y=c,c\in \left\{0,1\right\})$ can be readily estimated under the assumption that each component $x_{k}$ follows a Bernoulli distribution with success probability $p_{k,c}$. Specifically,

(1) $$\begin{array}{cc} P(x_{k}=1|y=c) & =p_{k,c}\\ P(x_{k}=0|y=c) & =1-p_{k,c} \end{array}$$

and the conditional probability is then given by

(2) $$\begin{array}{c} P\left(x_{k}|y=c\right)=p_{k,c}^{\;x_{k}}\;{\left(1-p_{k,c}^{\;x_{k}}\right)}^{\;1-x_{k}} \end{array}$$

#### 2.4.1. Training

During the BNBC training phase, the prior probability P(y=c) for each class *c* is computed as follows:

(3) $$\begin{array}{c} P\left(y=c\right)=\frac{1}{N}\sum_{i=1}^{N}1_{\left\{y^{i}=c\right\}} \end{array}$$

where $1_{\left\{y^{i}=c\right\}}$ is the indicator function:

(4) $$\begin{array}{c} 1_{\left\{y^{i}=c\right\}}=\left\{\begin{array}{cc} 1, & \mathrm{if}\;y^{i}=c\\ 0, & \mathrm{if}\;y^{i}\neq c \end{array}\right. \end{array}$$

For high-dimensional binary vectors, the probability of $p_{k,c}$ can be computed as

(5) $$\begin{array}{c} p_{k,c}=P\left(x_{k}=1|y=c\right)=\frac{\sum_{i=1}^{N}1_{\left\{y^{i}=c\right\}}x_{k}^{i}}{\sum_{i=1}^{N}1_{\left\{y^{i}=c\right\}}} \end{array}$$

#### 2.4.2. Testing

After training, the BNBC classifies each test sample by assigning it to the class *c* that maximizes the posterior probability. Each segment of the input signal is thereby labeled as either ictal or inter-ictal. Combining Equations (1) and (2) with Bayes’ theorem, the posterior probability of $\hat{X}$ belonging to class *c* can be written as

(6) $$\begin{array}{cc} \hat{Y} & =\arg\max_{c=0,1}P\left(y=c\right)\prod_{k=1}^{D}P\left({\hat{x}}_{k}|y=c\right)\\ \; & =\arg\max_{c=0,1}P\left(y=c\right)\prod_{k=1}^{D}p_{k,c}^{{\hat{x}}_{k}}{\left(1-p_{k,c}^{{\hat{x}}_{k}}\right)}^{\left(1-{\hat{x}}_{k}\right)}\\ \; & =\arg\max_{c=0,1}\left(\frac{1}{N}\sum_{i=1}^{N}1_{\left\{y^{i}=c\right\}}\right)\prod_{k=1}^{D}p_{k,c}^{{\hat{x}}_{k}}{\left(1-p_{k,c}^{{\hat{x}}_{k}}\right)}^{\left(1-{\hat{x}}_{k}\right)} \end{array}$$

#### 2.4.3. Lookup Table

In BNBC, an Lookup Table (LUT) mechanism is designed for both the training and testing phases in order to replace costly multiplications by integer additions. During training, the class-conditional probabilities $p_{k,c}$ are first estimated for each dimension *k* and class c∈{0,1}. The same probability values are then transformed and stored in the LUT and reused during testing. Starting from the Naive Bayes decision rule, the predicted label is obtained by

(7) $$\begin{array}{cc} \hat{Y} & =\arg\max_{c=0,1}\left\{\frac{1}{N}\sum_{i=1}^{N}1\left\{y_{i}=c\right\}\prod_{k=1}^{D}p_{k,c}^{x_{k}}{\left(1-p_{k,c}\right)}^{1-x_{k}}\right\}\\ \; & =\arg\max_{c=0,1}\left\{\log\left(\frac{1}{N}\sum_{i=1}^{N}1\left\{y_{i}=c\right\}\right)+\sum_{k=1}^{D}\left[x_{k}\log\left(p_{k,c}\right)+\left(1-x_{k}\right)\log\left(1-p_{k,c}\right)\right]\right\}, \end{array}$$

where $x_{k}\in \left\{0,1\right\}$ denotes the *k*-th component of the binary HD vector and $p_{k,c}$ is the probability that $x_{k}=1$ given class *c*. Equation (7) shows that classification only requires the evaluation of logarithms of probabilities and the accumulation of their (weighted) sums.

Because each $p_{k,c}$ lies in the range [0,1], we discretize these probabilities and store their negative logarithms as integers in an LUT. To this end, a scaled logarithmic transform is applied. The logarithm is multiplied by −128 for two reasons. First, the negative sign converts a maximization of the log-posterior into a minimization of a non-negative cost, which is convenient for integer accumulation. Second, the scaling factor $128=2^{7}$ provides a good compromise between numerical precision and hardware complexity. It yields sufficiently fine quantization of the log-probabilities while keeping the resulting integer values within a range that can be represented using a small fixed number of bits and implemented efficiently with integer adders and bit shifts.

Directly computing log(0) is undefined and very small probabilities may lead to numerical instability. To avoid this, we introduce a small offset and define the LUT function as

(8) $$\mathrm{LUT}\left(p\right)=\left\{\begin{array}{cc} -\log(p+0.005)\times 128, & p\leq 0.99,\\ 1, & p>0.99, \end{array}\right.$$

where *p* denotes a discretized probability value. The constant 0.005 corresponds to the midpoint of each probability bin of width 0.01; adding this offset avoids the singularity at p=0 and stabilizes the transform for very small probabilities, while still providing a good approximation to the continuous log-probability. For probabilities larger than 0.99, the corresponding negative log-probability becomes very small; we therefore clamp these values to 1 to avoid storing excessively small integers that would have negligible impact on the decision.

To maintain a simple and hardware-friendly implementation, we discretize the probability range [0,1] into 100 bins, which results in 100 distinct LUT entries. A finer discretization could in principle provide higher numerical precision, but would also increase the memory footprint of the LUT and the bit-width required to store each entry. The chosen resolution therefore reflects a trade-off between approximation accuracy and storage requirements. At the end of the training phase, all class-conditional probabilities $p_{k,c}$ are estimated and quantized to the nearest bin, and their corresponding integer values $\mathrm{LUT}(p_{k,c})$ are stored in a probability array. In addition, we pre-compute and store the negative log-posterior for each class, LUT(P(y=c)), to capture the prior distribution of class labels.

During testing, the HD input vector is processed by summing the appropriate LUT entries instead of evaluating products and logarithms. For each class *c*, we accumulate the integer costs associated with the active dimensions of the HD vector and add the stored class prior term LUT(P(y=c)). The class with the smallest accumulated cost is then selected as the predicted label, indicating whether the corresponding segment is ictal or inter-ictal. In this way, all expensive floating-point multiplications and logarithms are replaced by table lookups and integer additions, which are well suited for low-power embedded implementations. An example of the resulting LUT is given in Table 1.

### 2.5. Sliding Window Voting Detection

During the post-processing phase, predicted labels are aggregated into 5 s windows to generate the final ES detection results. A threshold $t_{p}$ is defined such that if the cumulative value within a window exceeds $t_{p}$, the window is classified as indicating a seizure. This classification is then used to calculate the detection delay associated with the actual seizure onset. For each patient, the latency is computed as the mean detection delay between the seizure onset point and the ES detection point.

## 3. Results

The proposed method is evaluated using the SWEC-ETHZ iEEG short-term dataset. Prior to conducting the experiments, the method is optimized for practical application scenarios. Initially, a BNBC is employed to classify high-dimensional vectors, with a focus on achieving higher sensitivity to meet practical requirements. We feed the high-dimensional vectors into the BNBC for learning and segment it into 0.5 s windows, with the dimensionality of the HD space set to 10,000. This approach, referred to as ’BNBC + 0.5 s + 10,000HD’, enhances both sensitivity and specificity; however, it also results in increased latency.

To address the latency issue, the 0.5 s window is replaced with a 0.25 s window, and the dimensionality of the HD space is reduced from 10,000 to 1000, resulting in the method termed ’BNBC + 0.25 s + 1000HD’. Furthermore, an LUT is added in both ’BNBC + 0.5 s + 10,000HD’ and ’BNBC + 0.25 s + 1000HD’, leading to the methods ’BNBC + 0.5 s + 10,000HD + LUT’ and ’BNBC + 0.25 s + 1000HD + LUT’. The number of seizures used for training and validation is summarized in Table 2. Overall and per-patient detection latencies are reported in Table 3. Across both one-shot and few-shot learning, our BNBC-based methods yield substantially lower average latency than Hamming-based HD baselines.

The results of the experiments are presented in Table 2. Four metrics are utilized to evaluate the method: latency, sensitivity, specificity, and accuracy. For each patient, latency is calculated as the mean detection delay between the seizure onset point and the ES detection point. Sensitivity measures the method’s ability to correctly identify ictal samples, reflecting its effectiveness in recognizing seizure periods; higher sensitivity indicates improved accuracy in ES detection and a lower rate of missed detections. Specificity assesses the method’s ability to correctly identify inter-ictal samples, with higher specificity corresponding to a lower false alarm rate and improved accuracy in detecting non-seizure periods. Accuracy represents the overall performance of the method in classifying ictal and inter-ictal samples, reflecting the proportion of correctly classified samples across all tasks. Compared to the results of [13,31], our method demonstrates improvements across all evaluation metrics. A detailed comparison of the different methods is provided in Table 2.

Notably, we observed that for some patients, the model obtained based on training with a single seizure sample (one-shot learning) instead outperformed the few-shot learning model trained with multiple seizure samples. This phenomenon is consistent with the findings of Burrello et al. [31], who noted that few-shot learning models may introduce more variability in seizure patterns or propagation paths in some cases, thereby increasing intra-class differences and leading to blurred classification boundaries.

Specifically, for patients with a clear, widespread, and stable seizure pattern, the one-shot learning model was able to learn highly representative features from a single sample to construct a concise and discriminative prototype vector. In contrast, a few-shot leaning model may introduce more inconsistent seizure types or local variations during training, which may lead to ‘feature dilution’ or ‘interference superposition’ in the prototype representation in high-dimensional hyperspace, and ultimately degrade the classification performance. Therefore, in practical applications, the adoption of the few-shot learning strategy should depend on individual patient characteristics, and the model performance does not always improve with the increase in the number of training samples.

Compared to Hamming-distance-based HD classifiers [13,31], the proposed BNBC+LUT framework exhibits superior performance and lower storage requirements under the same data and evaluation settings. On the one hand, out method improves sensitivity and specificity while stabilizing the average latency to within 4–5 s through a shorter window. On the other hand, the LUT replaces multiplication and logarithms with table lookup and integer addition, reducing the inference complexity and decreasing model storage from hundreds of KB to approximately 25–35 KB. This significantly outperforms previous Hamming distance classification methods.

### 3.1. One-Shot Learning

Compared to the Hamming methods [13,31], our methods achieved a sensitivity of approached 100%. The average latency was lowest in the BNBC + 0.5 s + 10,000HD method, recorded at 2.92 s. The BNBC + 0.25 s + 1000HD method exhibited an average latency of 3.15 s, with the lowest latencies observed in patients P4 and P5, while still maintaining stable detection performance. This indicates that our method achieves good performance even with a reduced dimensionality of the high-dimensional space. Furthermore, although the addition of the LUT resulted in a slight increase in average latency for the BNBC + 0.5 s + 10,000HD + LUT method, it remained low at 3.05 s. This demonstrates that the integration of the LUT does not significantly affect the latency.

### 3.2. Few-Shot Learning

As shown in Table 2, the BNBC + 0.5 s + 10,000HD method demonstrates the highest accuracy among the examined approaches. There is a strong performance observed in P9 and P10 (specificities of 92.31% and 97.68%, respectively). Overall, sensitivity exceeds 90% for all patients except P12. However, the average latency in few-shot learning is 6.31 s. Consequently, this method is most suitable for applications where classification stability is prioritized over low latency. By shortening the window size to 0.25 s and reducing the dimensionality of HD computing, this method further refines its performance for specific patients. Its mean sensitivity is 97.56% and the overall mean latency is reduced to 4.31 s, with P12 having the shortest latency of 0 s. After adding the LUT, the BNBC + 0.5 s + 10,000HD + LUT method shows a greater latency of 5.46 s on P15. Compared to the method without LUT, this method obtains higher sensitivity and lower specifity. Also, there is a reduction of 0.05 s in the average latency. The BNBC + 0.25 s + 1000HD + LUT performs well in P10 (specificity of 97.54%), and achieves approximately 100% sensitivity in some cases (e.g., P10 and P14), resulting in a highest mean sensitivity of 97.76% in few-shot learning. In addition, this method has a slight increase in average latecy to 5.65s. Overall, the BNBC + 0.5 s + 10,000HD method offers balanced performance and adaptability with sensitivity (97.99%) and specificity (95.9%). However, the latency of this method is 4.61 s, which is higher than BNBC + 0.25 s + 1000HD and BNBC + 0.25 s + 1000HD + LUT. The BNBC + 0.25 s + 1000HD has the lowest latency, which is 4.31 s. While the LUT adds and reduces the specificity of the method, it also elevates the latency of the model. However, it improves the overall sensitivity to 98.88%.

### 3.3. Storage Requirement

The storage requirements for the proposed method are evaluated using the first seizure of P1 as an example. P1 was implanted with 100 electrodes, so we can calculate the maximum storage requirement. This calculation below provides a detailed assessment of the storage requirement for the proposed BNBC + 0.25 s + 1000HD + LUT method. Given that the system employs 16-bit analog-to-digital conversion, each sample point requires 16 bits. Therefore, the storage for the LBP can be computed as follows:

(9) 16 bits×7 sampling points×100 electrodes=1400 bytes.

Additionally, storing 6 LBP values necessitates:

(10) 1bit×6LBPvalues×100electrodes=75bytes,

leading to a subtotal of 1475 bytes. Further storage is required for the LBP mapping matrix and the electrode mapping matrix, which contains 100 electrodes. Storing LBP mapping matrix (*C*) necessitates:

(11) 1000bits×64LBP=8000bytes.

Then, storing 6 LBP values necessitates:

(12) 1000bits×100electrodes=12500bytes.

while summation of XOR results consumes:

(13) 7bits×1000=875bytes,

For a 0.25 s duration, each sampling point requires at least 5 bits, contributing:

(14) 5bits×1000=625bytes,

Then, due to the nature of BNBC, we only need to store the histogram for one category. The storage for histograms in the BNBC training process is:

(15) 6bits×1000=750bytes,

while the LUT requires:

(16) $$\left(7\;\mathrm{bits}\times 1000+10\;\mathrm{bits}\times 100\;\mathrm{index}\right)/8=1000\;\mathrm{bytes}.$$

Approximately 10.5 additional bytes are required to store the results of the calculations and the post-processing threshold. Summing all these components yields a total storage requirement of 25,235.5 bytes (approximately 25.24 KB). It is important to note that the methods proposed in [13,31] do not fully account for the hardware requirements. Therefore, we calculated the storage needs for these methods using the same approach, with comparative results summarized in Table 2.

## 4. Conclusions

A new method for ES detection based on a BNBC for iEEG signals is proposed, achieving an average specificity of 93.09% and sensitivity of 98.88%, with the lowest average latency recorded at 4.31 s using one-shot and few-shot training strategies. This demonstrates the method’s effectiveness in detecting ES while minimizing missed detections and false alarms. For the first time, we combine BNBC with high-dimensional HD computation for ES detection, enabling a dimensionality reduction in high-dimensional vectors while improving overall sensitivity and latency. By segmenting the signals, features can be extracted from smaller segments, thereby reducing the average latency of ES detection. The robustness of our approach is validated through one-shot and few-shot learning experiments, highlighting its potential to provide timely and reliable ES detection for real-time monitoring and management of epilepsy, particularly in clinical and resource-limited settings.

Our approach combines an HD framework with BNBC, thereby reducing the effective dimension of the encoded vectors. Simultaneously, the overall performance surpasses that of HD classifiers based on Hamming distance in previous works. Furthermore, by segmenting the signal into short windows and extracting their LBP codes, we have reduced the average latency of ES detection. Within the classification component of the framework, we introduce LUT. By replacing floating-point multiplication and logarithmic operations with integer addition and table lookups, the entire framework becomes suitable for low-power, memory-constrained hardware platforms.

Despite these improvements, our work retains several limitations. Firstly, the experiments were conducted on a small sample size of 16 patients using short-term iEEG datasets. Secondly, we employed leave-one-seizure-out cross-validation without conducting cross-patient or cross-dataset validation. Furthermore, we have yet to realize a dedicated hardware prototype for power consumption and latency measurements.

Future work should focus on addressing these limitations. We plan to validate our approach on larger, more comprehensive datasets. This will include cross-patient and cross-dataset evaluations to better characterize robustness and generalization capabilities. Moreover, we aim to investigate adaptive learning and transfer learning strategies to reduce the volume of patient-specific data required for reliable performance. Finally, we shall explore concrete implementation approaches on neuromorphic or low-power embedded platforms, integrating LUT-based BNBC into real-time ES detection systems for clinical and resource-constrained environments.

## Figures and Tables

**Table 1:** Example construction of the Lookup Table (LUT) based on discretized probability values. The interval [0,1] is divided into bins of width 0.01, and each bin is mapped to an integer cost using LUT(p) in (8).

Index	Probability Range *p*	LUT(p)
1	[0.00,0.01]	884
2	[0.01,0.02]	577
3	[0.02,0.03]	495
4	[0.03,0.04]	445
⋮	⋮	⋮
99	[0.98,0.99]	2
100	[0.99,1.00]	1

**Table 2:** Comparison of sensitivity (Sens.), specificity (Spec.), and detection latency (*l*[*s*]) in one-shot and few-shot learning between different classifiers (BNBC, BNBC+LUT, and Hamming-distance-based HD methods [13,31]), signal windows (0.5 s and 0.25 s), and hyperdimensional vector sizes (1000HD, 10,000HD). ID denotes the patient identifier in the SWEC-ETHZ short-term iEEG dataset. N is the total number of annotated seizures for each patient, TrS is the number of seizures used for training in the one-shot or few-shot learning setting, and K is the number of folds used for cross-validation for each patient. *l*[*s*] represents the average detection latency measured from the clinical seizure onset. ’AVG’ indicates the average over all patients within each learning regime (one-shot or few-shot), whereas ’AVG(overall)’ denotes the average across all patients and both learning regimes. ’Accuracy’ gives the overall classification accuracy, and ’Storage (KB)’ reports the memory required to store the model parameters for each method. Boldface values highlight, for each patient, the best detection latency among the compared configurations, and in the ’Accuracy’ and ’Storage (KB)’ rows they mark the highest overall accuracy and the lowest storage requirement, respectively.

				BNBC						BNBC+LUT						Hamming					
ID	N	TrS	K	0.5 s + 10,000HD			0.25 s + 1000HD			0.5 s + 10,000HD			0.25 s + 1000HD			0.5 s + 10,000HD [13]			0.5 s + 10,000HD [31]		
				l[s]	Spec.[%]	Sens.[%]	l[s]	Spec.[%]	Sens. [%]	l[s]	Spec.[%]	Sens.[%]	l[s]	Spec.[%]	Sens.[%]	l[s]	Spec.[%]	Sens.[%]	l[s]	Spec.[%]	Sens.[%]
**One-shot Learning**																					
P2	4	1	4	4.31	100	100	**4.23**	100	100	4.88	100	100	5.2	100	100	15.1	100	100	10.4	100	100
P4	4	1	4	1.38	100	100	**1.06**	100	100	1.53	100	100	1.41	100	100	34.5	100	100	24.9	100	100
P5	6	1	6	2.01	99.96	100	**1.78**	100	100	1.83	99.88	100	2.12	100	100	20.9	100	100	19.3	99.42	100
P6	2	1	2	0.25	100	100	**0**	100	100	**0**	100	100	**0**	100	100	6.3	100	100	8	100	100
P8	3	1	3	3.06	100	100	3.06	99.26	100	**2.06**	100	100	3	99.26	100	13.2	100	100	12.9	100	100
P11	2	1	2	2.88	100	100	3.38	100	100	4.25	100	100	5.13	100	100	7	100	100	**2**	100	100
P13	2	1	2	2	100	100	2.81	100	100	**1.75**	100	100	2.88	100	100	10	100	100	7	100	100
P16	2	1	2	**7.5**	100	100	8.88	100	100	8.13	100	100	8.56	100	100	32.3	100	100	17.3	100	100
AVG	—	—	—	**2.92**	100	100	3.15	100	100	3.05	99.98	100	3.54	99.91	100	17.41	100	100	12.73	99.93	100
**Few-shot Learning**																					
P1	5	2	4	**1.1**	100	100	1.16	100	100	1.2	100	100	1.24	100	100	6.3	100	100	3.8	100	100
P15	9	2	8	5.63	100	100	5.9	100	100	**5.46**	100	100	6.04	100	100	36.4	100	100	22.6	93.91	100
P3	14	3	12	5.74	71.23	94.64	6.09	72.14	94.05	6.79	72.72	92.86	7.66	67.07	95.24	21.8	79.97	91.03	**4.1**	96.14	86.43
P7	7	3	5	12.4	83.87	100	4.66	75.23	100	10.90	79.55	100	1.24	67.51	100	5	49.9	88.57	**0.5**	89.27	90
P9	6	3	4	13.52	92.31	92.86	11.04	83.31	96.43	10.63	90.09	96.43	13.12	78.68	92.86	16.2	96.31	96.43	**0**	88.33	100
P10	13	3	11	4.68	97.68	100	4.5	97.56	100	4.09	96.56	100	4.57	97.54	100	3.9	98.41	94.41	**0.8**	97.44	94.52
P12	10	6	5	0.1	93.19	86	**0**	89.26	90	**0**	92.34	86	1.55	86.76	94	15.9	96.88	80	**0**	93.22	95
P14	10	4	7	**7.27**	96.14	94.29	10.39	93.36	100	9.51	95.68	97.14	9.76	92.64	100	10.5	95.94	85.71	7.9	99.53	76.17
AVG	—	—	—	6.31	91.8	95.97	5.47	88.86	97.56	6.07	90.87	96.55	5.65	86.27	97.76	14.5	89.68	92.02	**4.96**	94.73	92.77
AVG(Overall)	—	—	—	4.61	95.9	97.99	**4.31**	94.43	98.78	4.56	95.43	98.28	4.59	93.09	98.88	15.96	94.84	96.01	8.81	97.31	96.38
Accuracy	—	—	—	—	**96.94**		—	96.6		—	96.85		—	95.99		—	95.42		—	96.85	
Storage (KB)	—	—	—	342.74			35.47			239.11			**25.24**			242.74			633.59		

**Table 3:** Latency across BNBC/BNBC+LUT and Hamming baselines under one-shot, few-shot, and overall learning. The table reports per-patient average detection latency *l*[s]. In AVG rows, latency is summarized as mean ± SD across patients.

				BNBC		BNBC+LUT		Hamming [13]	Hamming [31]
ID	N	TrS	K	0.5 s + 10,000HD	0.25 s + 1000HD	0.5 s + 10,000HD	0.25 s + 1000HD	0.5 s + 10,000HD	0.5 s + 10,000HD
				l[s]	l[s]	l[s]	l[s]	l[s]	l[s]
**One-shot Learning**									
P2	4	1	4	4.31	4.23	4.88	5.2	15.1	10.4
P4	4	1	4	1.38	1.06	1.53	1.41	34.5	24.9
P5	6	1	6	2.01	1.78	1.83	2.12	20.9	19.3
P6	2	1	2	0.25	0	0	0	6.3	8
P8	3	1	3	3.06	3.06	2.06	3	13.2	12.9
P11	2	1	2	2.88	3.38	4.25	5.13	7	2
P13	2	1	2	2	2.81	1.75	2.88	10	7
P16	2	1	2	7.5	8.88	8.13	8.56	32.3	17.3
AVG	—	—	—	2.92 ± 2.21	3.15 ± 2.68	3.05 ± 2.57	3.54 ± 2.68	17.41 ± 10.93	12.73 ± 7.44
**Few-shot Learning**									
P1	5	2	4	1.1	1.16	1.20	1.24	6.3	3.8
P15	9	2	8	5.63	5.9	5.46	6.04	36.4	22.6
P3	14	3	12	5.74	6.09	6.79	7.66	21.8	4.1
P7	7	3	5	12.4	4.66	10.9	1.24	5	0.5
P9	6	3	4	13.52	11.04	10.63	13.12	16.2	0
P10	13	3	11	4.68	4.5	4.09	4.57	3.9	0.8
P12	10	6	5	0.1	0	0	1.55	15.9	0
P14	10	4	7	7.27	10.39	9.51	9.76	10.5	7.9
AVG	—	—	—	6.31 ± 4.77	5.47 ± 3.89	6.07 ± 4.16	5.65 ± 4.37	14.50 ± 10.85	4.96 ± 7.64
**Overall**									
AVG(Overall)	—	—	—	4.61 ± 3.99	4.31 ± 3.44	4.56 ± 3.69	4.59 ± 3.67	15.96 ± 10.62	8.84 ± 8.32

## Data Availability

The SWEC-ETHZ short-term iEEG dataset used in this study is publicly available at http://ieeg-swez.ethz.ch/ (accessed on 1 December 2025).

## References

[1] Bromfield E.B., Cavazos J.E., Sirven J.I. (2006). Basic mechanisms underlying seizures and epilepsy. An Introduction to Epilepsy [Internet].

[2] Kumar A., Maini K., Arya K., Sharma S. (2022). Simple Partial Seizure. StatPearls [Internet].

[3] Gavvala J.R., Schuele S.U. (2016). JAMA Patient Page: Epilepsy. JAMA.

[4] Czuczwar S.J. (2022). Epilepsy.

[5] Tran L.V., Tran H.M., Le T.M., Huynh T.T., Tran H.T., Dao S.V. (2022). Application of machine learning in epileptic seizure detection. Diagnostics.

[6] Sharmila A. (2018). Epilepsy detection from EEG signals: A review. J. Med. Eng. Technol..

[7] Acharya U.R., Molinari F., Sree S.V., Chattopadhyay S., Ng K.H., Suri J.S. (2012). Automated diagnosis of epileptic EEG using entropies. Biomed. Signal Process. Control.

[8] Jaiswal A.K., Banka H. (2018). Epileptic seizure detection in EEG signal using machine learning techniques. Australas. Phys. Eng. Sci. Med..

[9] Acharya U.R., Oh S.L., Hagiwara Y., Tan J.H., Adeli H., Subha D.P. (2018). Automated EEG-based screening of depression using deep convolutional neural network. Comput. Methods Programs Biomed..

[10] Cook M.J., O’Brien T.J., Berkovic S.F., Murphy M., Morokoff A., Fabinyi G., D’Souza W., Yerra R., Archer J., Litewka L. (2013). Prediction of seizure likelihood with a long-term, implanted seizure advisory system in patients with drug-resistant epilepsy: A first-in-man study. Lancet Neurol..

[11] Bekele M.W., Abera D.E., Hailemichael M.T., Dechasa K.Y., Fanos M.N., Getachew M.Y. Electroencephalography Data Denoising with Deep Neural Networks. Proceedings of the 2023 IEEE International Conference on Control, Electronics and Computer Technology (ICCECT).

[12] Karoly P.J., Freestone D.R., Boston R., Grayden D.B., Himes D., Leyde K., Seneviratne U., Berkovic S., O’Brien T., Cook M.J. (2016). Interictal spikes and epileptic seizures: Their relationship and underlying rhythmicity. Brain.

[13] Burrello A., Schindler K., Benini L., Rahimi A. (2019). Hyperdimensional computing with local binary patterns: One-shot learning of seizure onset and identification of ictogenic brain regions using short-time iEEG recordings. IEEE Trans. Biomed. Eng..

[14] Shoeibi A., Khodatars M., Ghassemi N., Jafari M., Moridian P., Alizadehsani R., Panahiazar M., Khozeimeh F., Zare A., Hosseini-Nejad H. (2021). Epileptic seizures detection using deep learning techniques: A review. Int. J. Environ. Res. Public Health.

[15] Avcu M.T., Zhang Z., Chan D.W.S. Seizure detection using least EEG channels by deep convolutional neural network. Proceedings of the ICASSP 2019-2019 IEEE international conference on acoustics, speech and signal processing (ICASSP).

[16] Hossain M.S., Amin S.U., Alsulaiman M., Muhammad G. (2019). Applying deep learning for epilepsy seizure detection and brain mapping visualization. ACM Trans. Multimed. Comput. Commun. Appl. (TOMM).

[17] Zuo R., Wei J., Li X., Li C., Zhao C., Ren Z., Liang Y., Geng X., Jiang C., Yang X. (2019). Automated detection of high-frequency oscillations in epilepsy based on a convolutional neural network. Front. Comput. Neurosci..

[18] Covert I.C., Krishnan B., Najm I., Zhan J., Shore M., Hixson J., Po M.J. Temporal graph convolutional networks for automatic seizure detection. Proceedings of the Machine Learning for Healthcare Conference, PMLR.

[19] Xu Y., Yang J., Ming W., Wang S., Sawan M. (2024). Shorter latency of real-time epileptic seizure detection via probabilistic prediction. Expert Syst. Appl..

[20] Hussein R., Palangi H., Ward R., Wang Z.J. (2018). Epileptic seizure detection: A deep learning approach. arXiv.

[21] Yuan Y., Xun G., Ma F., Suo Q., Xue H., Jia K., Zhang A. A novel channel-aware attention framework for multi-channel EEG seizure detection via multi-view deep learning. Proceedings of the 2018 IEEE EMBS international conference on biomedical & health informatics (BHI).

[22] Sahu R., Dash S.R., Cacha L.A., Poznanski R.R., Parida S. (2020). Epileptic seizure detection: A comparative study between deep and traditional machine learning techniques. J. Integr. Neurosci..

[23] Bayes T. (1958). An essay towards solving a problem in the doctrine of chances. Biometrika.

[24] Tzallas A.T., Tsipouras M.G., Fotiadis D.I. (2009). Epileptic seizure detection in EEGs using time–frequency analysis. IEEE Trans. Inf. Technol. Biomed..

[25] Sameer M., Gupta B. (2021). ROC analysis of EEG subbands for epileptic seizure detection using Naïve Bayes classifier. J. Mob. Multimed..

[26] Srihari P., Santosh V., Ganapathy S. (2023). An epileptic seizures diagnosis system using feature selection, fuzzy temporal Naive Bayes and T-CNN. Multimed. Tools Appl..

[27] Singh M., Jagyasi G., Garg M., Jain A., Gupta N., Kumar K., Kumar A. A Machine Learning Framework for Robust Epileptic Seizure Detection from EEG Sign. Proceedings of the 2023 3rd International Conference on Smart Generation Computing, Communication and Networking (SMART GENCON).

[28] Hasnaoui L.H., Djebbari A. Discrete Wavelet Transform and Sample Entropy-Based EEG Dimensionality Reduction for Electroencephalogram classification. Proceedings of the 2019 International Conference on Advanced Electrical Engineering (ICAEE).

[29] Shaik R., Goru H.K., Nadakuditi H., Vasamsetti G., Velagapudi B. A Comparative Study of Naive Bayes, LDA and Gradient Boosting Classifiers for Epileptic Seizure Detection Using Discrete Wavelet Transform. Proceedings of the 2023 14th International Conference on Computing Communication and Networking Technologies (ICCCNT).

[30] Burrello A., Schindler K., Benini L., Rahimi A. One-shot learning for iEEG seizure detection using end-to-end binary operations: Local binary patterns with hyperdimensional computing. Proceedings of the 2018 IEEE Biomedical Circuits and Systems Conference (BioCAS).

[31] Burrello A., Benatti S., Schindler K., Benini L., Rahimi A. (2020). An ensemble of hyperdimensional classifiers: Hardware-friendly short-latency seizure detection with automatic iEEG electrode selection. IEEE J. Biomed. Health Inform..

[32] Meng H., Appiah K., Hunter A., Dickinson P. FPGA implementation of Naive Bayes classifier for visual object recognition. Proceedings of the CVPR 2011 WORKSHOPS.

